# Nuclear localization of amyloid-β precursor protein-binding protein Fe65 is dependent on regulated intramembrane proteolysis

**DOI:** 10.1371/journal.pone.0173888

**Published:** 2017-03-21

**Authors:** Niina A. Koistinen, Anna K. Edlund, Preeti K. Menon, Elena V. Ivanova, Smaranda Bacanu, Kerstin Iverfeldt

**Affiliations:** Stockholm University, Department of Neurochemistry, Stockholm, Sweden; National Center for Geriatrics and Gerontology, JAPAN

## Abstract

Fe65 is an adaptor protein involved in both processing and signaling of the Alzheimer-associated amyloid-β precursor protein, APP. Here, the subcellular localization was further investigated using TAP-tagged Fe65 constructs expressed in human neuroblastoma cells. Our results indicate that PTB2 rather than the WW domain is important for the nuclear localization of Fe65. Electrophoretic mobility shift of Fe65 caused by phosphorylation was not detected in the nuclear fraction, suggesting that phosphorylation could restrict nuclear localization of Fe65. Furthermore, both ADAM10 and γ-secretase inhibitors decreased nuclear Fe65 in a similar way indicating an important role also of α-secretase in regulating nuclear translocation.

## Introduction

Processing of the Alzheimer disease (AD) associated amyloid-β (Aβ) precursor protein (APP) is mediated by at least three different proteases. In the amyloidogenic pathway, where the neurotoxic Aβ peptide is produced, the β-site APP cleaving enzyme BACE1 catalyzes ectodomain shedding [1]. This is followed by γ-secretase mediated cleavage [2] within the transmembrane (TM) domain leading to generation of two fragments: the APP intracellular domain (AICD) and the Aβ peptide. Abnormal production or insufficient removal followed by aggregation of Aβ is believed to be a key event in AD pathogenesis (reviewed by [3]). In the non-amyloidogenic α-secretase pathway, where formation of Aβ is precluded, shedding of APP leads to secretion of the neurotrophic/neuroprotective sAPPα fragment. In neurons, ADAM10 (a disintegrin and metallo-protease 10) has been identified as the main α-secretase [4,5]. As in the β-secretase pathway, the α-site cleavage is followed by formation of AICD mediated by the γ-secretase complex.

This type of proteolytic processing is referred to as regulated intramembrane proteolysis (RIP) [6,7]. RIP has emerged as an important mechanism involved in signal transduction during neuronal differentiation and plasticity. Signal transduction involves release of an ICD that can bind to proteins in the cytoplasm and/or nucleoplasm. APP/AICD interacts with a number of different proteins, including the adaptor protein Fe65. Through well-conserved protein-protein interaction domains: a tryptophan-tryptophan (WW) domain and two adjacent phosphotyrosine binding domains (PTB1 and PTB2), Fe65 can bind to various proteins forming multimeric complexes [8,9,10]. Fe65-PTB2 belongs to the Dab-like pY-independent NPXY-binding PTB domains, and has previously been shown to recognize the non-phosphorylated NPTY motif in APP [11,12,13]. Several studies indicate that Fe65 –APP interaction regulates APP processing.

In addition, nuclear Fe65 has been shown to be transcriptionally active together with AICD and the histone acetyltransferase Tip60 [14,15,16,17,18]. However, the mechanisms regulating Fe65 nuclear translocation are not fully understood. Binding to full-length APP seems to prevent Fe65 from translocating into the nucleus [19]. Furthermore, phosphorylation of either APP or Fe65 may promote Fe65 nuclear localization [20,21,22]. A recent study using a phospho-mimetic S^610^D Fe65 mutant indicates that phosphorylation within the PTB2 domain both disrupts the binding to APP and increases the nuclear localization [23]. On the other hand, in a study made by Jowsey and Blain, phosphomimetic mutants of Fe65 at S^228^ showed decreased transcriptional activity [24].

In this study we have further investigated the role of specific domains and phosphorylation of Fe65, as well as RIP, on Fe65 nuclear translocation. Our results suggest that PTB2 rather than the WW domain is important for the nuclear localization of Fe65. Our study also revealed that inhibition of α-secretase as well as phosphorylation can decrease Fe65 nuclear localization.

## Materials and methods

### Design and cloning of Fe65 Tandem Affinity Purification (TAP)-tagged constructs

A TAP tag was fused to human Fe65 by recombinant techniques. To generate a TAP-pcDNA3.1 vector, TAP tag sequence was PCR amplified from the pDS_LPCX-XB-TAP (ATCC ID:10326356) plasmid using following primers containing restriction sites for BamHI in the forward and XbaI in the reverse primer: forward 5’-TAATGGATCCGGAGGATCCTTGGAAAAGAG-3’, reverse 5’-TAATTCTAGATTAGGTTGACTTCCCCGCGGA-3’. The resulting PCR product was cloned into the pcDNA3.1(+) vector (Thermo Fisher Scientific). To generate TAP-tagged human Fe65 plasmids, p97Fe65 (1–710), p60Fe65 (261–710), ΔWWFe65 (328–710), and ΔPTB2Fe65 (1–520) sequences were amplified from pcDNA3.1-p97Fe65-myc (a gift from Dr. Qubai Hu), using the following primers containing restriction sites for BspTI in the forward and BamHI in the reverse primer: p97Fe65 forward 5’-TTAATCTTAAGGCCATGTCTGTTCCATCATCACTG-3’ and reverse 5’-TCTAGGATCCTGGGGTATGGGCCCCCAGC-3’, p60Fe65 forward 5’-TTAATCTTAAGGCCATGAGGGTCCAGGACACC-3’ and reverse 5’- TCTAGGATCCTGGGGTATGGGCCCCCAGC-3’, ΔWWFe65 forward 5’-TTAATCTTAAGGCCATGGAGCTGGGACTGAAG-3’, ΔPTB2Fe65 forward 5’-TTAATCTTAAGGCCATGTCTGTTCCATCATCACTG-3’ and reverse 5’-TCTAGGATCCTTTAGAGTGGTCCAGGGAGAGT-3’. The resulting PCR products were cloned into TAP-pcDNA3.1. p97Fe65, p60Fe65, ΔWWFe65 and ΔPTB2 were generated by mutating a STOP codon before the start of the TAP-tag of each Fe65 TAP-tagged construct. The Quick change II Site Directed Mutagenesis Kit (Agilent Technologies) was used according to the manufacturers’ protocol using the following primers: p97/p60/ΔWWFe65 forward 5’-GGCCCATACCCCATGATCCTTGGAAAA-3’ and reverse 5’-TTTTCCAAGGATCATGGGGTATGGGCC-3’, ΔPTB2Fe65 forward 5’-GGACCACTCTAAATGATCCTTGGAAAA-3’ and reverse 5’-TTTTCCAAGGATCATTTAGAGTGGTCC-3’.

### Design and cloning of APP constructs

To generate a wild type APP_695_ (APP_695_wt)-myc-pcDNA3.1 vector, a myc-tagged APP_695_wt sequence was amplified from APP-pcDNA1.1 plasmid described previously [25], using the following primers containing restriction sites for NheI in the forward and HindIII in the reverse primer, and a myc tag sequence in the reverse primer: forward 5’-TCTTGCTAGCGCCATGCTGCCCGGTTTGGCACTGCTCC-3’, reverse primer 5’-TATTCAAGCTTCTATTATCACAGATCTTCTTCAGAAATAAGTTTTTGTTCGTTCTGCATCTGCTCAAAG-3’. The resulting PCR product was cloned into the pcDNA3.1(+) vector (Thermo Fisher Scientific)

### Cell culture and treatment

SH-SY5Y (American Type Culture Collection) and SK-N-AS (European collection of cell cultures) human neuroblastoma cells were cultured as previously described [26]. The cells were maintained in a humidified 5% CO_2_ atmosphere at 37°C. For studies on nuclear localization of Fe65 upon secretase inhibition, SH-SY5Y cells were seeded at a density of 75 000 cells/cm^2^. 24 h after seeding, cells were grown for 24 h in the absence or presence of 5 μM N-[N-(3,5-Difluorophenacetyl)- L-alanyl]-S-phenylglycine t-butyl ester (DAPT, a γ-secretase inhibitor, Sigma), 2,5 μM GI254023X (an ADAM10 inhibitor, Sigma) and 5 μM Batimastat (a broad spectrum matrix metalloprotease inhibitor BB-94, Sigma). For studies on nuclear localization of TAP-tagged and untagged Fe65, SH-SY5Y cells were seeded at a density of 55 000 cells/cm^2^ and SK-N-AS cells at a density of 20 000 cells/cm^2^ prior transfection, whereas for interaction studies between Fe65-TAP and APP, SK-N-AS cells were seeded at a density of 35 000 cells/cm^2^ prior transfection. 24 h after seeding, SH-SY5Y and SK-N-AS cells were transfected with 5 μg plasmid DNA using X-tremegene HP (Roche). Briefly, DNA was mixed with 200 μl MEM and X-tremegene HP in a ratio of 1:3 (μg DNA:μl X-tremegene HP) and incubated at room temperature for 30 min. The mixture was then added to cells with freshly changed medium and incubated for 48 h for the TAP-tagged constructs and 72 h for the untagged constructs.

### Nuclear fractionation

Conditioned medium from SH-SY5Y cells was harvested and supplemented with Complete Protease Inhibitor (CPI) cocktail (Roche), centrifuged to remove cellular debris at 13 000 g for 30 min at 4°C before concentrating 5 times with a centrifugal filter (cut-off 30 kDa; VWR). Nuclear fractionation was performed as previously described [27]. The total protein concentration in the cytoplasmic and nuclear fractions were determined using bicinchoninic acid (BCA) protein assay kit (Thermo Fisher Scientific Inc.). The samples were mixed with sodium dodecyl sulfate (SDS) sample buffer prior to analysis by western blot.

### TAP-tag pull down

**Fig 1:**
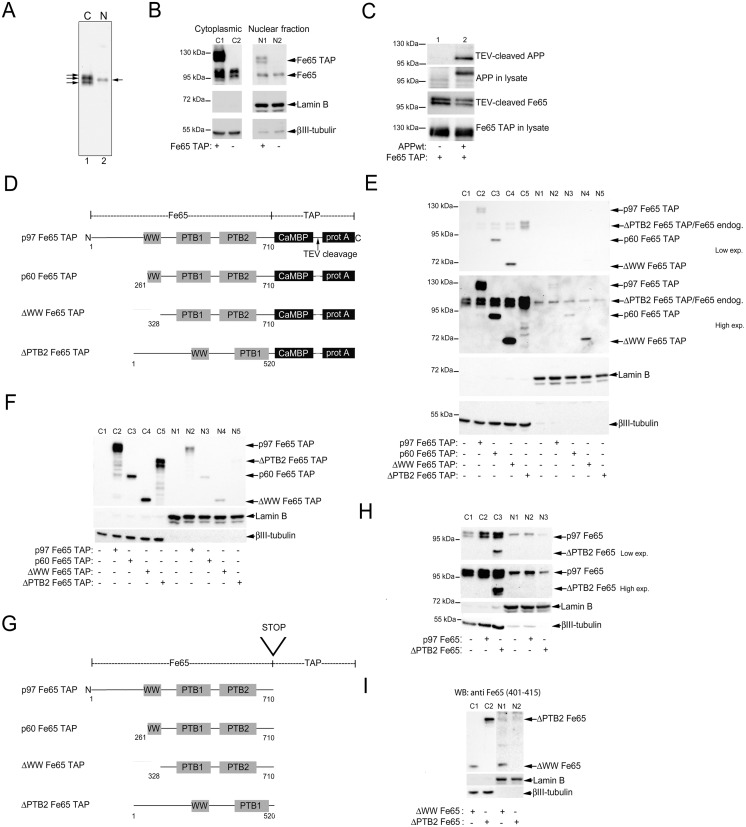
Subcellular distribution of overexpressed TAP-tagged and endogenously expressed Fe65. A. Representative western blot analysis of endogenously expressed Fe65 in cytoplasmic (C, lane 1) and nuclear (N, lane 2) fractions obtained from SH-SY5Y cells. B. Representative western blot analysis comparing levels of TAP-tagged Fe65 (p97Fe65 TAP) with endogenously expressed Fe65 in cytoplasmic (C) and nuclear (N) fractions obtained from SH-SY5Y cells. Lamin B and βIII-tubulin were used as markers for nucleus and cytoplasm, respectively. C. Representative western blot analysis of APP levels obtained after Fe65 TAP pull-down and TEV cleavage in SK-N-AS cells transfected with cDNA coding for Fe65 TAP and APPwt. D. Schematic illustration of Fe65 full-length (p97Fe65), the shorter isoform p60Fe65, and two deletion mutants fused to the TAP tag. E. Representative western blot analysis of cytoplasmic (C) and nuclear (N) fractions from SH-SY5Y cells overexpressing the different TAP-tagged Fe65 constructs. Two different exposure times are shown for optimal visualization of TAP-tagged Fe65 constructs in cytoplasmic and nuclear fractions, respectively. Note, the N-terminal Fe65 antibody (E-20) binds to the prot A sequence in the TAP tag leading to the visualization of the N-terminally truncated Fe65 constructs. F. Representative western blot analysis of cytoplasmic (C) and nuclear (N) fractions from SK-N-AS cells overexpressing the different TAP-tagged Fe65 constructs. Note, only secondary anti-goat IgG was used for visualization of TAP- tagged Fe65. G. Schematic illustration of Fe65 full-length (p97Fe65), the shorter isoform p60Fe65, and two deletion mutants lacking the TAP tag. H. Representative western blot analysis of cytoplasmic (C) and nuclear (N) fractions from SH-SY5Y cells overexpressing p97Fe65 and ΔPTB2Fe65. Two different exposure times are shown for optimal visualization of p97Fe65 and ΔPTB2Fe65 in cytoplasmic and nuclear fractions, respectively. I. Representative western blot analysis of cytoplasmic (C) and nuclear (N) fractions from SK-N-AS cells overexpressing ΔWWFe65 (C1 and N1) and ΔPTB2Fe65 (C2 and N2). For optimal visualization of Fe65 in cytoplasmic and nuclear fractions, the exposure time for nuclear fractions is 8 times longer than for the cytoplasmic fractions. Shown are representative images of three independent experiments.

TAP-tagged proteins together with their interacting partners can be recovered from cell extracts by a two-step affinity purification procedure [28,29]. In this study only the first purification step utilizing the IgG binding protein A (prot A) domain followed by TEV (tobacco etch virus) protease cleavage was employed (*cf*., Fig 1D). SK-N-AS cells were washed with 2x5ml of ice-cold 1x phosphate buffered saline (PBS) and lysed for 30 min at 4°C in NP-40 buffer (50 mM Tris-HCl pH 8,0, 150 mM NaCl, 1% NP-40, 1 mM EDTA pH 8,0) supplemented with 4 mM CPI. The cell lysates were centrifuged at 13 000 g for 15 min at 4°C before the total protein concentration of each sample was measured using BCA protein assay kit. 500 μg of total protein lysate of each sample was incubated with 60 μl of prewashed IgG Sepharose Fast Flow beads (GE Healthcare) for 3 h at 4°C. Next, the beads were washed with 2x500μl NP-40 lysis buffer followed by washing with 1x500 μl IPP50 TEV-buffer (10mM Tris-HCl, pH 8,0, 150 mM NaCl, 0,1% NP-40 and 0,5 mM EDTA). 100 μl IPP50 TEV-buffer and 10U TEV-protease (Life Technologies) were added to each sample and the beads were incubated at 4°C overnight on rotation. The TEV-cleaved samples (supernatants) were mixed with SDS sample buffer prior to analysis by western blot.

### Western blot analysis

10 μg of protein for detection of Fe65, APP, βIII-tubulin, and Lamin B in lysates or cell fractions, and 25 μl of concentrated conditioned cell medium for the detection of sAPPα, were subjected to electrophoresis on 8% Tris-glycine polyacrylamide gels. For the detection of TEV-cleaved Fe65-TAP and APP, 50 μl of each sample was loaded on 8% Tris-glycine polyacrylamide gels. Proteins were subsequently transferred to a nitrocellulose membrane (GE Healthcare) for 1h, 500 mA. Non-specific binding to the membranes was blocked by incubation in 5% non-fat dry milk prior to incubation with a primary antibody over night at 4°C, followed by secondary antibody incubation for 30 min at room temperature. Then immunoreactive proteins were detected using the enhanced chemiluminescence (ECL) system (SuperSignal West Dura Chemiluminescent Substrate, Thermo Scientific). Antibody concentrations used were 1:3000 for E-20 (directed against the N-terminus of Fe65, Santa Cruz), 1:1000 for A3860 (directed against amino acids 401–415 of Fe65, Sigma) 1:2000 for 6E10 (to detect sAPPα, BioLegend), 1:3000 for CT-APP (Sigma), 1:3000 for βIII-tubulin (Abcam), 1:1000 for Lamin B (Abcam). Note, TAP-tagged Fe65 was detected by most of the antibodies, also by the secondary anti-goat IgG alone due to the protein A domain. The secondary antibody concentrations were 1:5000 for horseradish peroxidase-coupled anti-goat IgG (Abcam), anti-rabbit IgG (GE Healthcare) and anti-mouse IgG (GE Healthcare).

### Analysis of Fe65 phosphorylation

Cytoplasmic and nuclear fractions were incubated with or without alkaline phosphatase (FastAP, Thermo Scientific) according to the manufacturer’s protocol. Reactions were stopped by addition of SDS sample buffer. Proteins in reaction mixtures were analyzed on 8% Tris-glycine polyacrylamide gels.

### Immunofluorescence

For immunofluorescent labeling of TAP-tagged constructs, SK-N-AS cells were seeded out on glass coverslips in a 6-well plate at a density of 40 000 cells/cm^2^ prior transfection. 24 h after seeding, the cells were transfected with 1 μg plasmid DNA using X-tremegene HP (Roche) for 48 h in a ratio of 1:3 (μg DNA:μl X-tremegene HP). After 48 h incubation, transfected cells were washed with 2x2mL of ice cold 1xPBS. Subsequently the cells were fixed and permeabilized with -20°C methanol for 5 min followed by washing with 3x2mL of ice cold 1xPBS. Cells were then blocked in 1x Tris-buffered saline (TBS, 137mM NaCl, 2,7mM KCl, 25mM Tris-Base, pH 7,4) containing 5% bovine serum albumin (BSA) and 1% Tween-20 at room temperature for 1 h. Thereafter, cells were incubated with an Alexa Fluor 488-conjugated donkey anti-goat IgG antibody (Invitrogen, A11055) and DRAQ5 (for DNA staining) at concentrations 1:2000 and 1:5000 respectively, for 1 h at room temperature. Note, TAP-tagged Fe65 was directly detected by the anti-goat antibody due to the protein A domain. The cells were washed 3x500μL/coverslip in 1xTBS containing 1% Tween-20 before mounting on glass slides with Fluoromount-G (SouthernBiotech, 0100–01). Subcellular distribution of immunostained TAP-tagged Fe65 was determined using an imaging system with a Nipcov spinning disc (CSU-22) on a Zeiss Axiovert 200.

## Results

### Endogenous and TAP-tagged full length Fe65 are detected in nuclear fractions and the PTB2 domain seems important for the nuclear localization

Fe65-APP/AICD interaction has been observed both in the cytosol and in the nucleus. Here, we wanted to further investigate the nuclear localization of Fe65, since this is essential for transcriptional activity. Western blot analysis after subcellular fractionation of SH-SY5Y cells showed that endogenously expressed Fe65 was present both in the cytoplasmic and nuclear fractions (Fig 1A). We estimated the nuclear fractions to contain approx. 4% of the total cellular Fe65. Next, we wanted to investigate if TAP-tagged full length Fe65 (p97Fe65-TAP) was translocated into the nucleus (Fig 1B). The TAP tag is designed for isolation and analysis of protein complexes and can be used as a tool to study protein-protein interactions. The TAP tag comprises two domains; the IgG binding protein A (prot A) domain and a calmodulin binding domain (CaBD), separated by a TEV (tobacco etch virus) protease cleavage site (Fig 1D). Full-length Fe65 (also designated p97Fe65) was fused to the TAP tag (Fig 1D). In addition, an APP695wt-myc (APPwt) cDNA construct was made. Initially, SK-N-AS cells, which do not express detectable levels of endogenous APP695, were cotransfected with p97Fe65-TAP cDNA and cDNA encoding APPwt. TAP tag containing complexes were pulled down using IgG coated beads followed by TEV protease elution. Western blot analysis revealed that APPwt was successfully pulled down together with p97Fe65-TAP demonstrating that the TAP tag does not interfere with the APP-Fe65 interaction (Fig 1C). As shown in Fig 1B and 1F, p97Fe65-TAP was also detected in the nuclear fraction of both SH-SY5Y and SK-N-AS cells. This is of interest since it opens up the possibility to use the TAP tag two step purification method to identify interacting partners in complex with Fe65 both in the cytoplasm and in the nucleus by mass spectrometry.

Several studies have reported that an N-terminally truncated form of Fe65 designated as p60Fe65 exists. This shorter isoform has been shown to be upregulated in the brains of p97Fe65-specific knock out mice [30]. p60Fe65 was also shown by immunofluorescence studies to be localized in the nucleus when overexpressed [31]. The WW domain of Fe65 is considered to be important for nuclear localization of Fe65, and for AICD dependent transcriptional activity [15,19], and p60Fe65 lacks part of this domain. We wanted to further investigate p60Fe65 and how deletion of the total WW domain, or of the APP binding PTB2 domain affects the nuclear translocation. The N-terminally truncated 60 kDa isoform (p60Fe65, amino acids 261–710), a deletion mutant lacking the N-terminus including the WW domain (ΔWWFe65, amino acids 328–710), and a deletion mutant lacking the C-terminus including the PTB2 domain (ΔPTB2Fe65, amino acids 1–520) of Fe65 were fused to the TAP tag, as illustrated in Fig 1D. Overexpression of the different cDNA constructs in both SH-SY5Y and SK-N-AS cells followed by subcellular fractionation and western blot analysis revealed the presence of p97Fe65, p60FE65 and ΔWWFe65 in the nuclear fraction, whereas ΔPTB2Fe65 was barely or not at all detected (Fig 1E and 1F).

To rule out that the large TAP tag is involved in the inhibition of ΔPTB2Fe65 nuclear localization, the TAP tag was removed by introducing a STOP codon before the start of the TAP tag (Fig 1G). Initially, p97Fe65 and ΔPTB2Fe65 without the TAP tag was overexpressed in SH-SY5Y cells. Subcellular fractionation and western blot analysis revealed that ΔPTB2 was not recovered in the nuclear fraction (Fig 1H). Similar results were obtained from SK-N-AS cells overexpressing ΔPTB2Fe65 and ΔWWFe65 (Fig 1I). Subcellular fractionation and western blot analysis revealed that ΔWWFe65 was detected in the nuclear fraction, whereas ΔPTB2Fe65 was not (Fig 1I).

**Fig 2:**
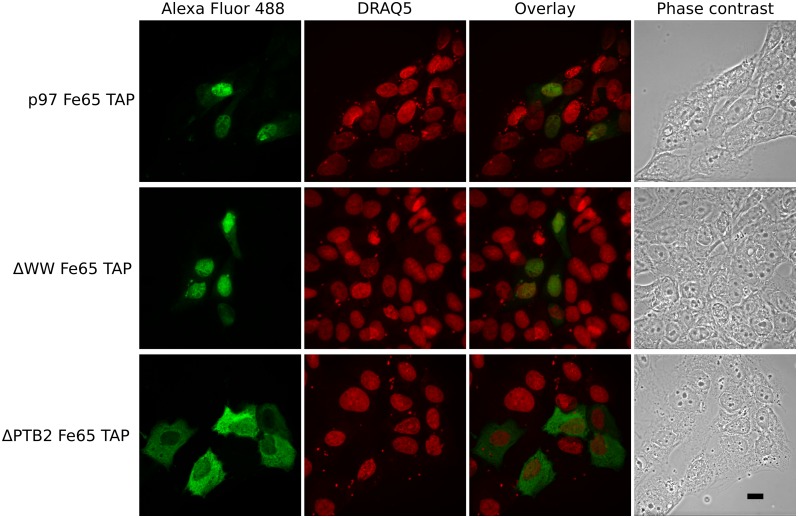
ΔPTB2Fe65 differ in its subcellular localization from p97Fe65 and ΔWWFe65. Representative immunofluorescence microscopy images of SK-N-AS cells expressing p97Fe65-TAP (top row), ΔWWFe65-TAP (middle row), or ΔPTB2Fe65-TAP (bottom row). Fe65-TAP constructs were labeled with Alexa Fluor 488-conjugated anti-goat antibody. Cell nuclei were stained with DRAQ5. p97Fe65-TAP and ΔWWFe65-TAP were mainly localized in the nucleus whereas ΔPTB2Fe65-TAP was preferentially distributed to the cytoplasm. Note, TAP-tagged Fe65 was detected by the secondary anti-goat antibody alone due to the protein A domain. Scale bar 10 μM. Shown are representative images of three independent experiments.

To verify the results received from western blot analysis, p97Fe65-, ΔWWFe65- and ΔPTB2Fe65-TAP overexpressing SK-N-AS cells were also subject to immunofluorescence studies. Immunostaining revealed that p97Fe65 and ΔWWFe65 were mainly localized in the cell nuclei whereas ΔPTB2Fe65 was largely excluded from the nuclei and was preferentially localized in the cytoplasm (Fig 2).

### Fe65 nuclear localization is dependent on RIP and phosphorylation

**Fig 3:**
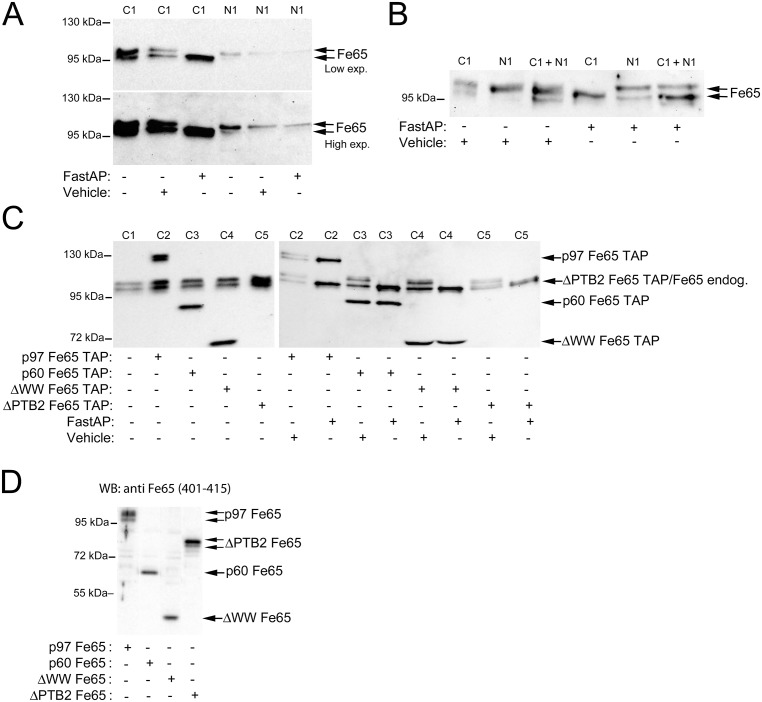
Fe65 electrophoretic mobility shift due to phosphorylation is not detected in the nuclear fraction. A. Western blot analysis of Fe65 in cytoplasmic (C1) and nuclear (N1) fractions after incubation in the absence or presence of alkaline phosphatase. Two different exposure times are shown for optimal visualization of Fe65 in the different fractions. B. Western blot analysis of Fe65 in cytoplasmic (C1), nuclear (N1) and cytoplasmic and nuclear mixed (C1 + N1) fractions after incubation in the absence or presence of alkaline phosphatase. C. Western blot analysis showing the effect of alkaline phosphatase treatment of cytoplasmic fractions on endogenous Fe65 and p97Fe65-TAP (C2), p60Fe65-TAP (C3), or the deletion mutants ΔWWFe65-TAP (C4) and ΔPTB2Fe65-TAP. D. Representative western blot analysis of lysates from SK-N-AS cells overexpressing the different Fe65 constructs lacking the TAP tag. The results are from the same blot. Different exposure times are shown for optimal visualization.

When analyzed by western blot, Fe65 migrates as several bands (Fig 1A), and here we wanted to further investigate which of these that can be translocated into the nucleus. Both the cytoplasmic and nuclear fractions were subjected to alkaline phosphatase treatment. As our previous results show [32], the electrophoretic mobility shift of endogenous Fe65 occurring in the cytoplasmic fraction was abolished by alkaline phosphatase treatment, resulting in the appearance of Fe65 as a single band (Fig 3A and 3B). In contrast, treatment of the nuclear fraction with alkaline phosphatase did not give rise to any significant mobility shift of the Fe65 band (Fig 3A and 3B). This indicates that these phosphorylated forms of Fe65 are preferentially localized in the cytoplasm of SH-SY5Y cells.

When overexpressing the different TAP-tagged Fe65 deletion mutants, we discovered that two of the mutants, p60Fe65 and ΔWWFe65, migrated as one band, whereas p97Fe65 and ΔPTB2Fe65 migrated as several bands according to western blot analysis (Figs 1F and 3C). To investigate the occurrence of phosphorylated forms of the different TAP-tagged Fe65 mutant proteins, the cytoplasmic fractions of SH-SY5Y cells overexpressing these constructs were subjected to alkaline phosphatase treatment. Western blot analysis revealed that the heterogeneity of p97Fe65 and ΔPTB2Fe65 bands was due to phosphorylation (Fig 3C). On the other hand, alkaline phosphatase treatment did not have any effect on the bands corresponding to p60Fe65 and ΔWWFe65 suggesting that these forms of Fe65 are not phosphorylated in SH-SY5Y cells. To show that the TAP tag did not have any effect on the migration of the bands, Fe65 deletion mutants lacking the TAP tag (Fig 1G) were overexpressed in SK-N-AS cells. Subsequent western blot analysis of cell lysates revealed that the TAP tag did not affect the migration of the bands (Fig 3D). Thus, our results indicate that phosphorylation(s) within the first N-terminal 261 amino acids of Fe65 gives rise to the observed electrophoretic mobility shift.

**Fig 4:**
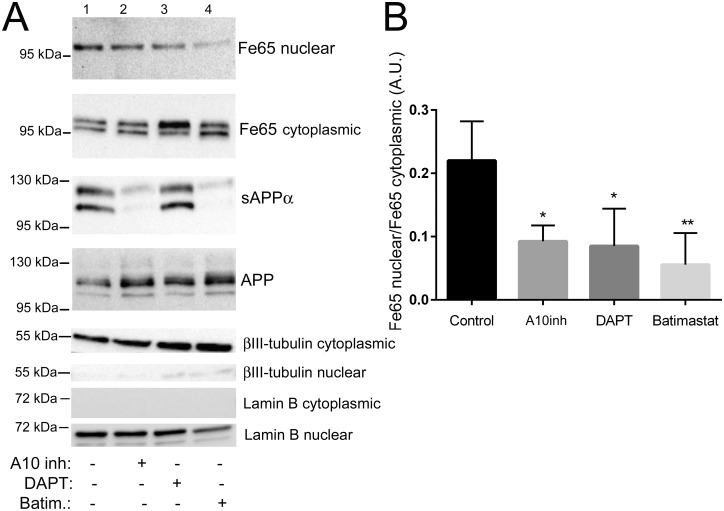
Inhibition of ADAM10 or γ-secretase decreases the abundance of endogenous Fe65 in the nuclear fraction. A. Representative western blot analysis showing the levels of endogenously expressed Fe65 in the cytoplasmic and nuclear fraction of SH-SY5Y cells after treatment with the ADAM10 inhibitor GI254023X (A10inh), the γ-secretase inhibitor DAPT and the non-selective ADAM inhibitor Batimastat. The levels of sAPPα in the medium and APP in the cell lysate are shown for comparison. Lamin B and βIII-tubulin were used as markers for nucleus and cytoplasm, respectively. B. Relative abundance of Fe65 in the nuclear fraction (presented as in Fig 2C). *P<0.05, **P<0.01, compared to control, n = 4.

As Fe65 is found in the nucleus and has been shown to be involved in transcriptional regulation, especially together with AICD, we wanted to further investigate how the inhibition of α- and γ-secretase affects the nuclear localization of Fe65 in SH-SY5Y cells. As expected, from previous studies showing that the nuclear AICD is generated through the β-secretase pathway [33,34], γ-secretase inhibition by DAPT decreased the nuclear localization of Fe65 (Fig 4A and 4B). We also observed that DAPT induced an electrophoretic mobility shift of Fe65 which we previously showed was due to phosphorylation [32]. Both the ADAM10 specific inhibitor GI254023X and the broad spectrum matrix metalloprotease inhibitor Batimastat significantly reduced the secretion of sAPPα into the medium as expected, since ADAM10 has been shown to be the major α-secretase of APP in neuronal cells [4,5]. Most interestingly, α-secretase inhibition also resulted in decreased levels of nuclear Fe65 to a similar degree as DAPT (Fig 4A and 4B). Both the ADAM10 specific inhibitor GI254023X and the broad spectrum matrix metalloprotease inhibitor significantly decreased the levels of nuclear Fe65.

## Discussion

The brain enriched adaptor protein Fe65 is a multifaceted protein acting on diverse cellular functions, such as transcriptional regulation and DNA repair. Several Fe65 binding proteins have been identified, of which APP is the most studied due to its role in AD. Fe65 interaction with APP or its fragment AICD has been shown to occur both in the cytosol and in the nucleus. This interaction occurs between the PTB2 domain of Fe65 and the highly conserved YENPTY motif of APP. Mutating the Y^687^ in the YENPTY^687^ motif to Ala is sufficient to completely abolish the interaction between Fe65 and APP (unpublished data).

Fe65 has been shown to play a role in APP/AICD-dependent effects on transcription [15,35]. The WW domain and both PTB1 and PTB2 domains of Fe65 were shown to be required for stimulation of APP/AICD dependent transactivation [15]. It should be noted that a study by Yang *et al*. [36] showed that overexpression of p97Fe65 with Gal4 fused to the C-terminus (p97Fe65-Gal4) was sufficient to increase transcriptional activity. Transactivation was observed in several different cell lines, including HEK293 and the neuroblastoma cell line SK-N-SH, but was not detected in COS cells. Interestingly, the study further showed that when Gal4 was fused to p97Fe65a2, an Fe65 isoform with an altered C-terminal region lacking part of the APP binding site [37], transactivation was reduced by approx. 90% [36]. In addition, the deletion of the 55 C-terminal residues of Fe65 resulted in translocation of Fe65 from the nucleus to the cytosol, results that correlate with our data indicating that PTB2 rather than the WW domain seems important for the nuclear localization of Fe65. Transactivation of Gal4 fused to the N-terminus of p97Fe65 has also been studied showing no significant stimulation of reporter gene expression [35,36,38]. However, strong transcriptional activity could be induced by the deletion of both PTB domains of Fe65 [35,38]. Based on these results it was suggested that the WW domain is responsible for Fe65 nuclear localization and functions as a transcriptional activator. Cao and Südhof [35] also proposed that the lack of transactivation of p97Fe65 was due to that Fe65 adopts a closed conformation via intramolecular interactions, between the WW and PTB domains, inactivating its transcriptional activity.

The mechanisms behind Fe65 translocation to the nucleus as well as which domains are important are not completely understood. Fluorescence microscopy studies have indicated that the WW domain is required for nuclear translocation [19]. Another study using subcellular fractionation suggested that the spacer region between WW and PTB1 domains (amino acids 292 to 364) is needed [39]. In addition, it was shown that either the region N-terminal of the WW domain (191–253) or the region containing the PTB2 domain (510–655) is required [39]. Our results show that TAP-tagged full length Fe65 (1–710) and p60Fe65 (261–710) are detected in the nuclear fractions. These results are in agreement with immunofluorescent studies which show nuclear localization of the 60kDa Fe65 isoform [31]. Noteworthy, we also detected the TAP-tagged Fe65ΔWW (328–710) in the nucleus both by western blot and by immunostaining, whereas TAP-tagged Fe65ΔPTB2 (1–520) was barely detected or not detectable. From our results the PTB2 rather than the WW domain seems important for the nuclear localization of Fe65 in both SH-SY5Y and SK-N-AS cells. It has been shown that Fe65 binds through its PTB1 domain to the histone acetyltransferase Tip60 [15]. Hass and Yankner have proposed that APP recruits Tip60 to the membrane and induces complex formation with Fe65 [40]. Fe65 may be translocated to the nucleus together with Tip60 which harbors both potential nuclear localization and export signals. It might be possible that in the absence of the Fe65-PTB2 domain the APP/Fe65/Tip60 complex formation is disturbed.

The function of Fe65 may also be regulated directly by phosphorylation. Our previous studies have shown that phosphorylation of Fe65 is upregulated by neuronal differentiation [32]. Phosphorylation commonly induces an electrophoretic mobility shift when analyzed by western blot. After subcellular fractionation of SH-SY5Y cells we observed that Fe65 in the nuclear fraction appeared as a single band. As expected, the heterogeneity of the Fe65 bands in the cytoplasmic fraction was due to phosphorylation. However, alkaline phosphatase treatment of the nuclear fraction had no effect on the electrophoretic mobility of Fe65. Thus, we conclude that the phosphorylated forms of Fe65 (causing the electrophoretic mobility shift) are preferentially localized outside the nucleus.

p60Fe65 and ΔWWFe65 migrated as single bands, that were not affected by treatment with alkaline phosphatase, in contrast to the TAP-tagged full length Fe65 and ΔPTB2. This suggests that the phosphorylation of Fe65 (that causes the electrophoretic mobility shift) occurs mainly in the N-terminal part of the protein. Previously it was shown, in a mutational study, that removal of the first 191 N-terminal amino acids of Fe65 does not have any effect on the heterogeneity of the Fe65 bands. However, deleting the N-terminal 356 amino acids, which includes the WW domain, resulted in the appearance of only a single band [39]. Together with our results this suggests that the phosphorylation causing the shift occurs within the amino acid sequence between 192 and 260. This amino acid sequence contains several putative phosphorylation sites. Interestingly, phosphorylation at residue S^228^ has been reported to inhibit APP-Fe65 mediated gene transcription since phosphomimetic mutants (S^228^D and S^228^E) showed decreased transcriptional activity, whereas when mutated to Ala the opposite effect was observed [24]. It should also be noted that phosphorylation of at least two sites within the PTB2 domain has been associated with increased nuclear localization/transcriptional activity, namely S^610^ [23] and Y^547^ [22]. Phosphorylation at these sites may not occur to a large extent in the cells used in our study, or may not cause a detectable mobility shift.

In addition to the role of APP promoting Fe65 nuclear localization (as proposed by Hass and Yankner [40]) APP has also been suggested to directly prevent this action by anchoring Fe65 to the membrane. APP is one of the membrane-bound receptors proposed to function as a proteolysis-dependent latent transcription factors undergoing RIP. Here we wanted to investigate the role of RIP in regulation of Fe65 nuclear localization. We observed that there was a significant decrease in the levels of Fe65 in the nuclear fractions in response to either α- or γ-secretase inhibition. It may seem surprising that blocking the α-secretase pathway strongly affects Fe65 nuclear localization, since previous studies indicate that APP/AICD dependent transcription occurs predominantly through the β-secretase (BACE1) pathway [33,34]. However, it has been proposed that AICD and Fe65 translocate into the nucleus independently [41]. Therefore, Fe65 released from the membrane by the α-secretase pathway may not be linked to APP dependent transcription. However, it cannot be excluded that the results reflect that APP is only one of the membrane bound interaction partners of Fe65, and that other signaling pathways involving Fe65 are also processed by ADAM10.

In conclusion, the effect of deleting the Fe65 PTB2 domain and blocking the α-secretase supports: 1) the importance for anchoring Fe65 to the plasma membrane, 2) that the release from the plasma membrane to a large degree is dependent on ADAM10 cleavage, and 3) that nuclear translocation, at least partly, is independent of AICD. Finally, phosphorylation of Fe65 in the N-terminal domain appears to inhibit nuclear translocation and thereby most likely its effect on transcription.
